# Translations

**Published:** 1848-10-01

**Authors:** 


### CRIMINAL STATISTICS OF FRANCE.

The 'Moniteur' publishes the report of the Minister of Justice on the administration of the laws in criminal cases during the year 1846. It appears from this report, that during that year, 6908 individuals had been tried on criminal charges. Of those 1878 were accused of crimes against the person, and 5030 of crimes against property. In comparing the number of criminals with that of the year 1845, the number accused of crimes against the person had diminished by 173, and that of those accused of crimes against property had increased by 396; the increase being 3 per cent. This increase is regarded by the Minister of Justice as inconsiderable, when the scarcity of food during the latter months of the year 1846 is considered. This increase, moreover, is shown to be less than that which appeared from 1826 to 1844. The number of those accused of heinous crimes in the year 1846 remained stationary as compared with 1845. Amongst the crimes against property there are three which present an increase over those of the year 1845—viz. incendiary fires, fraudulent bankruptcy, and qualified robberies. The 6908 individuals accused of crime in the year 1846 consisted of 5743 men and 1165 women. The accused were aged as follows:—1199, or one-sixth, under 21 years; 2204 from 21 to 30 years; 1686 from 30 to 40 years; 1111 from 40 to 50 years; 455 from 50 to 60 years; and 253 above 60 years. Amongst the minors there were 72 who had not attained their sixteenth year. They were tried by the Court of Assize either because they had accomplices older than themselves, or that their crimes are punished with death. The unmarried accused formed, as they do every year, more than half of the whole number. There were 2749 married accused, and 325 widowers or widows. The two-thirds of the accused—viz. 4563, were tried in the departments where they were born, 1426 resided there, but were born elsewhere, and 919 were strangers by birth and by domicile. Amongst the latter 302 were not born on the French soil, 3996 inhabited rural communes, 2646 urban communes, and 266 lived a vagabond life. Each of the professions into which society is classed supplied its contingent to the accused. There were 4702 field labourers, 496 tradesmen or clerks, 322 carters or mariners, 143 hotel or lodging-house keepers, and 517 house servants; 349 of the accused enjoyed an independent income, and 289 possessed no visible means of existence. Of 100 accused in the year 1846, more than the half—viz., 52, knew not either how to read or write, and the remaining 48 were but imperfectly instructed. The proportional number of illiterate accused is greater amongst those accused of crimes against the person than amongst those accused of crimes against property. Of the 6908 individuals tried during the year 1846, 2269 were acquitted, 1835 were condemned to degrading punishments, and 2774 to correctional punishment, and of 30 under the age of 21 years, who were declared to have acted without discernment, 24 were sentenced to remain in houses of correction, and six were sent home to their parents. Forty convicts were executed in the year 1846. The capital punishment was commuted in favour of 11 to hard labour for life, and the 52nd committed suicide after his appeal was registered. The courts of assize of the 13 departments were called on to try 11 individuals for offences of the periodical press, 20 for offences of the press not periodical, and 9 for political offences; 26 were acquitted, 13 were condemned to pay a fine and to suffer imprisonment, and one to pay a fine only. Amongst the 6908 accused, who were tried in the year 1846 before the Court of Assize, 1781 had been previously convicted, 148 to hard labour, 104 to solitary confinement, 601 to more than a year's imprisonment, 893 to one year or less, and 35 to pay a fine only. The number of accidental deaths during the year 1846 amounted to 7558; of which 3861 were caused by drowning, 624 were crushed to death by carriages, and 45 were caused by railroad accidents. It appears that the number of suicides is increasing every year. There were 2814 in the year 1841, 2866 in 1842. They increased to 3084 in 1845, and to 3102 in 1846; amongst the latter were 773 women. Amongst the suicides were 27 children, from 10 to 15 years of age, 139 between 16 and 21 years, 443 from 21 to 30 years, 1214 from 30 to 50 years, 513 from 50 to 60 years, 403 from 60 to 70 years, 209 from 70 to 80 years, and 51 above 80 years. Suicides are more frequent in spring and summer than in winter and autumn. The months of June, July, and August, produced 940. Those of March, April, and May, 904. Those of September, October, and November, 654; and those of December, January, and February, 604. Strangulation and suspension were the means most frequently employed by the suicides of 1846 to terminate their existence; 1077 had recourse to it; 1036 drowned themselves; 222 suffocated themselves with charcoal, and 429 use fire-arms. Amongst the suicides recorded in the year 1846, more than a quarter, viz., 888, were caused by insanity. The others are various. Physical sufferings and domestic unhappiness, pecuniary embarrassment, and the fear of distress, are the most frequent.

